# Betablockers for haemodynamically stable acute myocarditis

**DOI:** 10.1186/1532-429X-16-S1-P277

**Published:** 2014-01-16

**Authors:** Lorenzo Monti, Claudio Moro, Lucia Occhi, Veronica Lisignoli, Giuseppe Iacuitti, Daniela Pini, Barbara Nardi, Maddalena Lettino, Luca Balzarini

**Affiliations:** 1 Radiology, Humanitas Research Hospital, Rozzano(MI), Italy; 2 Cardiology, Humanitas Research Hospital, Rozzano, MI, Italy; 3 Cardiology, P.O. Desio, Desio, MB, Italy

## Background

No therapy is actually recommended for hemodynamically stable acute myocarditis patients. However, most patients are discharged on empiric therapy. We sought to evaluate any eventual effect of medical therapy on left ventricular ejection fraction (LVEF) at f.u.

## Methods

We analyzed CMR data form 44 patients hospitalized from our E.D. with acute myocarditis (diagnostic CMR performed after a mean of 5 days after admission, f.u. CMR after a mean of 5,4 months).

## Results

baseline LVEF was similar (p = 0.2) between ACE e BB groups, 61.9 ± 5% and 58,3 ± 7% respectively. DUAL group had a lower mean LVEF of 54 ± 11%. At follow up, LVEF was unchanged in ACE group ( from 61.9 to 61.2%), and improved in BB group, from 58 to 63% (p = 0,04). In DUAL group LVEF improved in a similar extent from 54 to 59% (p 0,01). Myocardial T2 STIR edema, significantly decreased at f.u. in all groups. All the remaining CMR parameters had non-significant modification from baseline to f.u.; LGE mass showed borderline significance toward reduction (p = 0.066)

## Conclusions

With the limitation of the small sample size of our series of hemodynamically stable acute myocarditis, we observed a greater improvement of LVEF at 6 months in pts treated with betablocker therapy, irrespective of concomitants ACEi therapy.

## Funding

None.

